# Introducing a new severity specifier for schizophrenia: conceptual framework and clinical implications

**DOI:** 10.47626/1516-4446-2024-3722

**Published:** 2024-11-25

**Authors:** Ary Gadelha, Renan M. Biokino, Pedro Lorencetti, Nicolas A. Crossley, Daniela Bordini, Raffael Massuda

**Affiliations:** 1 Departamento de Psiquiatria, Escola Paulista de Medicina, Universidade Federal de São Paulo (EPM/UNIFESP), São Paulo, SP, Brazil; 2 Programa de Esquizofrenia, EPM/UNIFESP, São Paulo, SP, Brazil; 3 Laboratório Interdisciplinar de Neurociências Clínicas, Departamento de Psiquiatria, EPM/UNIFESP, São Paulo, SP, Brazil; 4 Departamento de Psiquiatría, Pontificia Universidad Católica de Chile, Santiago, Chile; 5 Ambulatório de Autismo, UNIFESP, São Paulo, SP, Brazil; 6 Departamento de Psiquiatria, Universidade Federal do Paraná, Curitiba, PR, Brazil

**Keywords:** Psychosis, functioning, specifier, schizophrenia, severity

## Abstract

Patients with schizophrenia represent a group heterogeneous in clinical presentation and severity. Although severity has been operationalized in different ways, mostly on a gradient between symptom severity and functional impairment, such approaches are limited in capturing real-world functioning. We suggest adopting the severity model proposed by DSM-5 for autism spectrum disorders. The model defines three levels of severity based on the support required, directly addressing two barriers from previous models: real-world functioning and ease of implementation. Our adapted model includes three new features: first, this severity specifier is global, rather than for each symptom domain; second, the centrality of occupational functioning is emphasized to define the level of support; third, we propose a 1-month timeframe for severity appraisal, standardizing the assessment process. Considering practical utility, we indicate how severity assessment can guide clinical practice towards rehabilitation. Additionally, we outline operational definitions for severity and functioning in schizophrenia, aligned with the premises of our model. Finally, we acknowledge potential limitations, the most relevant being the need for empirical validation. The model is presented to foster discussion. Additional studies will follow to investigate inter-rater reliability and convergent validation with standard measures of symptom and functioning severity.

## Introduction

Patients with schizophrenia exhibit substantial variability in disorder severity. While some individuals can maintain symptom and functional stability over several years, leading productive and fulfilling lives, a significant portion will experience varying degrees of impairment, with some facing persistent functional disability.^1^ Furthermore, in the same patient, symptom severity can fluctuate throughout the course of the disorder.^2^ Although clinicians consider severity when making treatment decisions,^3^ a standardized classification of severity has yet to be integrated into everyday clinical practice.

We propose the introduction of a severity specifier for schizophrenia based on functionality, measured by the level of support needed for real-world functioning. Initially, we will examine the methods employed in assessing the severity of schizophrenia, aiming to identify both advantages and limitations that can aid in its practical implementation. Subsequently, we will introduce our proposed model, providing a comprehensive outline of its operationalization criteria. Finally, we will address constraints and detail the subsequent steps required for validation.

## Background: severity rating in schizophrenia

Traditionally, the severity of schizophrenia is evaluated on the basis of symptom intensity, assessed by standardized instruments such as the Positive and Negative Syndrome Scale (PANSS) and the Brief Psychiatric Rating Scale (BPRS). These scales were designed to systematically measure symptoms and the response to medication in individuals with schizophrenia.^4^-^6^ Moreover, tools widely used to assess functioning in research include symptoms to convey a rating. In the Global Assessment of Functioning (GAF), individuals are rated on a scale of 0 to 100, all centered around the presence of symptoms.^7^ In the original Clinical Global Impression (CGI) scale, seven levels are defined, also combining symptoms and functioning.^8^ While CGI includes social functioning in its criteria, the specific grading of functioning remains ambiguously defined.^9^ A schizophrenia-adapted CGI, CGI-Schizophrenia (CGI-SCH), defines five domains, four for symptoms, and one for global severity, thus also encompassing symptoms and severity in the same construct.^10^

Diagnostic and treatment clinical guidelines also approach severity as symptom intensity. In DSM-5, the severity specifier identifies eight symptomatic dimensions, each with a scale from 0 to 4: hallucinations, delusions, disorganized speech, abnormal psychomotor behavior, negative symptoms, impaired cognition, depression, and mania.^11^ The recent DSM-5 revision maintained the same severity criteria. In ICD-11, specifiers allow for characterization of the course – first, multiple, continuous – and the degree of symptom intensity in the current episode, but there is no proper disorder severity specifier. It is still possible to define intensity for six symptom domains.^12^ The treatment guidelines issued by the Royal Australian and New Zealand College of Psychiatrists (RANZCP) mention severity, mostly linked to symptom intensity.^13^ Interestingly, on two occasions, the guidelines suggest that disorder severity must be considered in decision making, but do not clarify how to assess it. Finally, the National Institute for Health and Care Excellence (NICE) guidelines mention severity twice – first referring to symptoms, second to psychotic episode severity – without ever defining it.^14^

More recently, there has been growing emphasis on assessing functional outcomes independently of symptom severity. The Social and Occupational Functioning Assessment Scale (SOFAS), derived from the GAF, was introduced in the DSM-IV-TR to evaluate social functioning, with 10 anchoring levels of impairment severity. To address the specific needs of schizophrenia research, tools like the Personal and Social Performance (PSP) scale were developed. The PSP is widely used in schizophrenia trials, often as a secondary outcome. Despite its brevity, inconsistencies in its application have been observed, highlighting the importance of proper training.^15^ Other instruments have been used in schizophrenia research as well. The University of California, San Diego Performance-Based Skills Assessment (UCSD UPSA) is a performance-based assessment comprising five subdomains: planning recreational activities, finance, communication, mobility, and house management.^16^ UPSA is designed to assess cognitive impairment indirectly and requires training and a specific set of material for its application. The Functioning Assessment Short Test (FAST) is an interview-rated scale that has good psychometric properties and measures six specific areas of functioning: autonomy, occupational functioning, cognitive functioning, financial issues, interpersonal relationships, and leisure. Its 24 different items make administration time-consuming, and proper training is again required.^17^

In essence, severity measurements typically refer to instruments directed towards symptoms or functioning. Functioning is assessed together with symptoms to define severity in some instruments, but independently in others. However, social, real-life performance, and even cognition may be assessed as functioning domains. Furthermore, available instruments utilize different evaluation methods, including self-reporting, clinician assessment, and performance-based tasks, and each instrument typically employs a different scoring system, conveying either a single or multiple scores. Even within instruments providing a single final score, several domains, items, or anchoring levels are considered, ranging from five to 24 in the instruments listed above.

## Looking for a better proxy for severity

As detailed above, symptom intensity, functioning, and cognitive impairment have all been used as overlapping concepts to account for severity. Nonetheless, symptoms alone do not provide a complete overview of functioning. Pedersen & Karterud^18^ showed that GAF scores exhibited a stronger correlation with functioning rather than symptomatology. In a recent investigation, both negative symptoms and cognitive performance emerged as significant predictors of functioning. However, this model only elucidated 30% of variance in functioning. Overall, while all symptom dimensions hold predictive value for functioning, their collective explanatory power remains limited.^19^,^20^

The gap observed between assessments and daily life may be better understood by acknowledging the difference between the concepts of functional capacity (ability to perform well in an optimal situation) and real-world functioning (what a patient actually does).^21^,^22^ Although in a slightly different approach, this distinction is also referred to as competence versus performance.^23^ Several variables can explain the discrepancy between capacity and real-world performance, like characteristics of the disorder, financial resources, and the patient’s social context. Many patients may perform well or seem more preserved during an appointment or a formal assessment, while performing badly in real-world settings.^22^

Assessments of severity should align more closely with real-life behavior. Additionally, measuring real-life performance should capture higher-order behaviors rather than categorizing them into specific domains. A recent study illustrated that real-life behaviors explain variance in functioning better than symptoms and cognitive performance.^24^

This perspective is consistent with the definition outlined in the International Classification of Functioning (ICF), which underscores that an individual’s functioning is influenced not only by disease or personal attributes but also by interactions with their environment.^25^,^26^ Considering that disability is not person-centered but contextually determined, this definition emphasizes that the level of support can influence the level of disability.^26^ More than merely providing a definition, such an approach proposes a course of action. Thus, assessing the required level of support is more in line with contemporary definitions of functioning and rehabilitation than solely relying on symptom severity or standard questionnaires that address functioning, which are usually better at capturing capacity than real-life performance.

## Definitions, instruments, and clinical differences of functionality

The absence of consensus on definitions poses additional challenges. For instance, “functioning” lacks a singular definition, and various “subcategories” remain inadequately operationalized, including social, psychosocial, and occupational aspects.

We outlined operational definitions for severity and functioning in schizophrenia following the premises summarized above. Severity in the context of schizophrenia represents the extent of impairment observed in overall real-life behavior. Functioning should be characterized as the degree of performance, globally or within specific domains, exhibited in real-world behavior. While there may be some overlap, these concepts differ, with one emphasizing impairment and the other highlighting performance, either generally or within specific domains.

The trade-off between capturing disorder complexity and being practical represents another barrier. Current instruments, even simple ones, often encompass multiple domains in their assessments. This is intended to better capture phenomenon granularity, which can be extremely valuable for research purposes and specialist clinicians, but creates difficulties for translation to practice in community settings. Take for instance the CGI-SCH, an instrument known for its user-friendly nature: it has five dimensions, each further divided into seven levels, resulting in thousands of different possible combinations. These myriad categories significantly complicate the decision-making process from data analysis to operationalization of recommendations. The more complex the instrument, the more training it will require, preventing its widespread use in clinical settings and undermining its reliability.

Cultural factors pose an extra hurdle for ensuring reliable measurements across diverse countries. For instance, the FAST criteria categorize living with parents after 20 years of age as a marker of poor functioning. However, this perception is clearly questionable in most Latin societies (at least in Latin American culture). Therefore, while a lack of consensus on definitions poses challenges, overly stringent definitions can also introduce bias when comparing data across distinct cultural backgrounds.

In conclusion, a severity classification to be used in clinical settings must be brief, with few categories, be based on a single observable real-world behavior or dimension, not require extensive training, and account for cultural diversity.

## A new severity specifier for schizophrenia

Specifiers are defined in the DSM-5 as extensions to a diagnosis that clarify the course, severity, or special features of a disorder, enabling counselors to select more effective treatments.^11^ We advocate that the solution to the severity classification of schizophrenia can be found in the DSM-5 itself, although in another chapter. The severity staging level adopted for autism spectrum disorder (ASD) is user-friendly and acknowledges the idea that severity may vary based on the context or fluctuate over time.^11^

The criteria for ASD underwent changes from DSM-IV-TR to DSM-5. Key amendments included replacing subtypes like Asperger’s syndrome and childhood disintegrative disorder (Heller syndrome) with a unified category; grouping symptoms from three to two dimensions; and introducing a new severity specifier.^11^ This specifier applies to each of the two symptom dimensions and is based on the level of support required for performing daily activities.^27^

This definition can be understood as a proxy to real-life functioning and a possible answer to some of the challenges outlined above.

Despite some authors’ criticism regarding the severity specifier, it gained quick acceptance among clinicians and patients’ families, demonstrating practicality and insightfulness.^28^ In Box 1, we propose a similar model as an additional definition of severity in schizophrenia to reflect the extent of impairment observed in overall real-life behavior.

The adapted criteria outlined herein differ somewhat from the original version proposed for ASDs. Initially, we suggest a single severity measurement for the disorder as a whole rather than specific dimensions. The rationale is simplicity, aiming for practical use in clinical settings. Second, recognizing heterogeneity across functioning domains, we adopted occupational functioning as the core feature for operationalization. Occupational functioning serves as the target for rehabilitation programs in the first episode of psychosis.^29^ Third, we suggest a 1-month timeframe for assessment, whereas the criteria for ASD do not specify a timeframe. Our justification is to establish criteria that are easy to use and enhance reliability in clinical settings.

**Figure 1:**
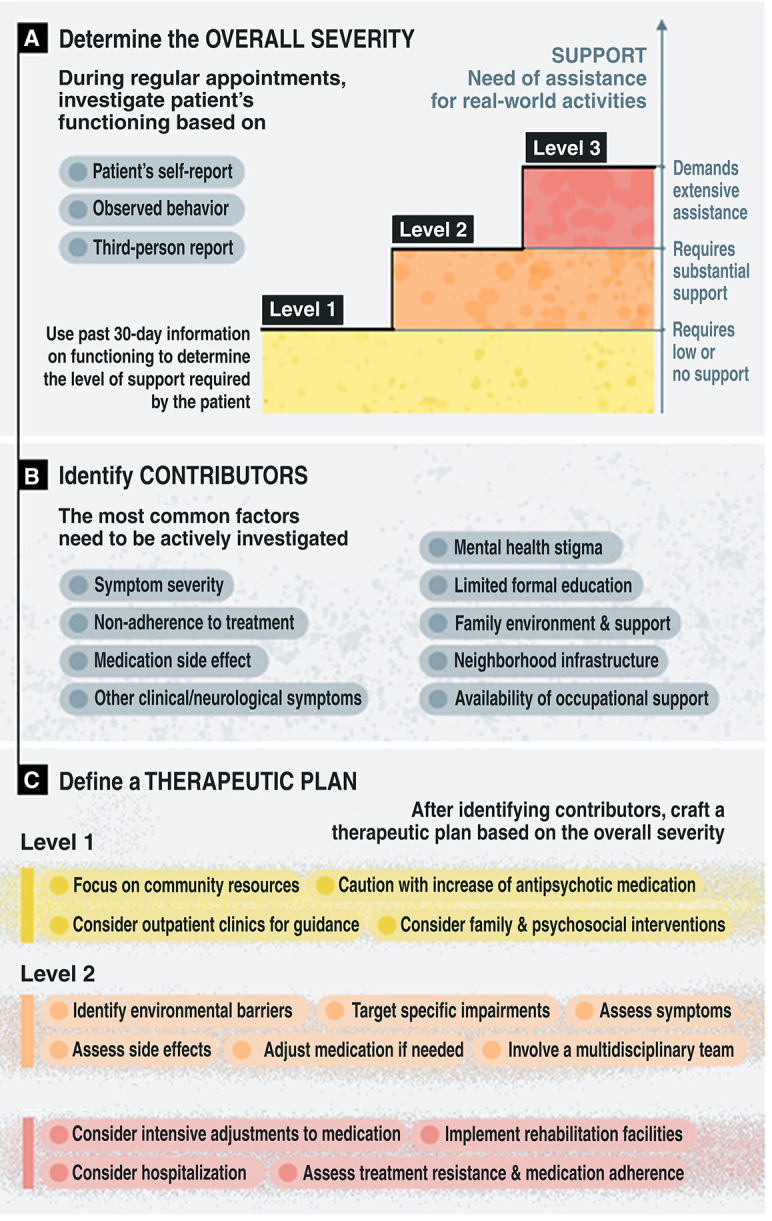
Suggested systematic approach.

A genuine concern in the field is that relying on a single global score may overlook deficits in specific domains.^30^ We recognize this risk. Indeed, finding a single measure, scale, or questionnaire that is both practical and comprehensive is challenging. To address this, it is crucial to understand that we are proposing a process, not just a single measure. Starting with assessment of severity level as the initial step, followed by two additional steps (Figure 1), would allow for a more nuanced approach. Subsequent steps can then incorporate more detailed instruments or assessments. For a thorough evaluation of functioning instruments, we recommend the review conducted by Searle et al.^31^

### Assessing severity during a regular appointment

The severity assessment should be conducted at each appointment, drawing information from multiple sources: the patient’s self-report, behavior observed during the interview, and third-person reports about the patient’s behavior. While all sources can be used, if not all are available, the definition is to be guided by the best available information. The assessment should mirror the flow of a regular appointment, starting with open-ended questions about the patient’s well-being and current activities. Subsequently, specific questions addressing suggested functional domains can be posed. Questions related to functioning should emphasize measurable behaviors, using practical examples. For example, if a patient mentions having friends, inquire about their most recent gathering; if they reference a job, delve into the specifics of their role. We recommend assessing the frequency of behaviors over the past month. A shorter period may not allow for reliable pattern detection, while a longer timeframe increases the likelihood of memory biases, potentially influencing the accuracy of data provided by patients or other informants.

To assess the support, two variables should be observed: initiative, i.e., the ability to initiate behavior without someone’s encouragement, and support itself, characterized by the assistance of another person. The level of support can range from verbal encouragement to the need to perform an activity together throughout its course. The need for support and encouragement should be assessed by asking specific questions and, again, should also consider the behavior observed during the interview and all other available sources of information.

As outlined above, we propose a comprehensive approach to severity, targeting overall real-life behavior as indicated by the support level required. Focus on concrete aspects of daily life is recommended over subjective self-reports. The delineation between the three levels is determined by functioning in occupational activities. In addition, the recommended assessment timeframe is 1 month.

### How to use the severity specifier to design an intervention plan

After defining the severity level, the underlying causes must be investigated. The most usual factors should be actively addressed:

- Symptom severity
- Non-adherence to treatment
- Side effects of antipsychotic medications
- Side effects of other medications
- Family environment and support
- Neighborhood infrastructure
- Stigma (at various levels)
- Availability of occupational support
- Other clinical or neurological symptoms

After employing this two-level approach – assessing both overall severity and contributors to severity – the subsequent step involves drafting a therapeutic plan. The degree of severity must be carefully considered to determine the scope and context of recommended interventions.

For example, at Level 1, any increases in antipsychotic medications should be approached with caution, weighing the benefits of improving symptom control against the burden on functioning imposed by adverse effects. Support should primarily tap into community resources rather than relying solely on healthcare services. In this context, enlisting tutors or professionals for guidance within outpatient clinics can be greatly beneficial. Additionally, family-based and psychoeducational interventions should not be overlooked.

Transitioning to Level 2, implementation of more targeted interventions focused on addressing the specific factors contributing to impairment becomes crucial. A thorough assessment of symptom intensity and a careful consideration of medication side effects should be undertaken. If necessary, adjustments should be made to the medication regimen. Environmental barriers are to be identified and verified with the patient and their family. A plan should be developed to coordinate actions among a multidisciplinary mental health team.

At Level 3, particularly when symptoms are significantly prominent, a more intensive adjustment of antipsychotic therapy may be warranted. Treatment-resistant schizophrenia and antipsychotic adherence need to be objectively assessed. Support in the form of day-hospitalization or full hospitalization may be required; referral to mental health rehabilitation facilities and intensive support should be arranged.

Once the first assessment has been completed and an initial intervention plan defined, follow-up assessments can reveal new challenges. For example, if a patient returns for their subsequent appointment exhibiting a noticeable increase in severity and a decline in functioning compared to the previous period, even in the absence of more severe symptoms, this signals the need for a more thorough and comprehensive assessment. Questions arise: Did the patient adhere to the prescribed medication regimen? Did stressors or difficulties in performing daily activities disrupt the patient’s stability? It is worth noting that functioning and other behavioral characteristics have already been established as more sensitive indicators of relapse than symptoms.^32^

## Discussion

In the context outlined above, we find several strengths in our proposal:

- Ease of implementation – It does not require the use of structured questionnaires or extensive training.
- Focus on general functioning and support needs – It directs clinicians towards assessing occupational functioning and the need for support, rather than focusing solely on specific symptoms or functioning domains as treatment targets. This supplements assessment of symptoms and bolsters rehabilitation efforts.
- Defines outcomes for mental health services – It establishes a measurable outcome for health systems, namely, the reduction of functional severity over time.
- Potential to reduce stigma – It has the potential to mitigate stigma by recognizing that some patients may exhibit mild or even no impairment. Patients with better outcomes often do not identify with descriptions of more severe cases, and therefore tend to reject the diagnosis; even some psychiatrists struggle to diagnose schizophrenia in patients with milder symptoms. Acknowledging different levels of severity and recognizing patients with better prognoses can help both patients and their families accept the disorder more readily.
- Short-term approach – As it is short-term in nature, there is no need for extensive historical assessments or future prognosis.
- Leverages a successful strategy – It builds upon an approach that has already demonstrated positive outcomes in the context of autism.^33^
- Makes customized decision-making easier – It may enable more individualized decision-making, particularly in cases where empirical data regarding prognosis or treatment options are available.

The definition of level of functioning carries two significant implications. First, it enables a thorough examination of the underlying causes of impairment. Second, it determines the type of support required and the most suitable setting for its implementation. An additional noteworthy outcome of this staging system is that success is defined by the transition from Level 2 to Level 1, a milestone that is informative not only for mental health teams but also for patients and their families.

The limitations of this proposed model must also be acknowledged:

- Decision-making is individualized – This severity model does not dictate or preclude treatment decisions. It serves as a parameter to assist a clinician’s assessment. It is essential to view the severity specifier as one factor among many that assist clinicians in conceiving a therapeutic plan, rather than the sole determining factor.
- Dependence on support data availability – Effective categorization of the need for support relies on data regarding how patients function in various settings, such as at home or in social/work environments. Challenges may arise when patients have difficulty providing accurate information or when data sources are unreliable. This challenge can be partially addressed by asking specific questions about how patients carry out their tasks, aiming for consistency and a true understanding of their actions. In cases where available data are insufficient or unreliable, caution should be exercised in using severity staging to inform clinical judgment.
- Subjective appraisal in categorization – The outlined categorization model still involves a level of subjective judgment. Hence, additional studies should be conducted to ascertain whether the provided description allows for good inter-rater reliability.
- Occupational functioning as the defining element – Functioning may vary across different domains. While a more comprehensive assessment is necessary to capture nuanced variations, this diversity can complicate definition of a single level of severity. Occupational functioning is easily and objectively verifiable, requires a higher level of functioning, and is more sensitive to deficits in other areas; it represents a core domain essential for autonomy in real-life settings. Nevertheless, further studies will be necessary to confirm whether this assumption overestimates severity and to recommend the best approach.

The proposed severity specifier does not replace existing staging models, instruments for assessment of functioning, or the dimensional approach outlined in the latest DSM-5 and ICD-11 criteria for schizophrenia. Instead, we see it as offering a supplementary framework. Furthermore, it has the potential to encourage systematic evaluation of functioning and severity during routine appointments, ultimately enhancing overall clinical assessment of each patient.

Our proposal was built on recent changes in the classification of ASD. It is important to note that the Intellectual Developmental Disorders category in the ICD-11 and their corresponding diagnoses in the DSM-5 also adopt a similar approach, which has been widely used in clinical practice for many years. While there are some differences between these classifications and our proposal, this only reinforces the potential for an easy-to-use severity classification system.^11^,^12^

It is important to clearly state that this is a proposal and should not be immediately applied in clinical practice. The main limitation is the need for testing in real-world settings to better understand the clinical utility of the proposed model. After this step, we believe our specifier could be added to schizophrenia diagnosis in DSM-5 and ICD-11. Each model is only as good as the next one with better clinical utility to overcome it. Our initial aim with this detailed proposal is to include other researchers and clinicians in the discussion, which should certainly improve it in many ways and lead to well-designed studies to assess it.

## Disclosure

AG has been a consultant/advisor and has received honoraria from Daiichi-Sankyo, Janssen, Critália, Lundbeck, Ache, and Boehringer Ingelheim. RM has been a consultant/advisor and has received honoraria from Daiichi-Sankyo, Janssen, and Boehringer Ingelheim. The other authors report no conflicts of interest.

## Figures and Tables

**Box 1:** Description of each level of severity

Level	Support required	Description
Level 1 Mild	Low or none (e.g., requiring someone for assistance in real-world activities)	In the past month, patients required minimal to no support in terms of functioning, handling most activities independently for most of the time.
		The endorsed category should reflect predominance in occupational activities (educational or professional activities appropriate for the individual’s age and cultural context).
		Patient can engage in occupational activities with minimal support or encouragement.
Level 2 Moderate	Substantial (e.g., someone must be present throughout an entire activity or day period)	In the past month, support was required for most activities, impacting functioning across all or nearly all domains.
		Encouragement alone is insufficient for occupational activities, but the patient is able to perform tasks under supervision.
		Less demanding activities such as domestic affairs, self-care, and family living are generally preserved.
Level 3 Severe	Very substantial	Over the past month, intensive assistance was required for all activities of daily living, i.e., the individual was unable to participate in work or study, required guidance for self-care, and experienced significant impact on social relationships, even with substantial support.
		Patients at risk to themselves or others also fall into this category.
		Need for hospitalization due to a relapse is a likely indicator of Level 3 severity.
